# Identifying the primary site of pathogenesis in amyotrophic lateral sclerosis – vulnerability of lower motor neurons to proximal excitotoxicity

**DOI:** 10.1242/dmm.018606

**Published:** 2015-03

**Authors:** Catherine A. Blizzard, Katherine A. Southam, Edgar Dawkins, Katherine E. Lewis, Anna E. King, Jayden A. Clark, Tracey C. Dickson

**Affiliations:** 1 Menzies Research Institute Tasmania, University of Tasmania, Hobart, TAS 7000, Australia; 2 Wicking Dementia Research and Education Centre, University of Tasmania, Hobart, TAS 7000, Australia

**Keywords:** Motor neuron disease, Amyotrophic lateral sclerosis, Excitotoxicity, Lower motor neuron, Excitotoxin exposure

## Abstract

There is a desperate need for targeted therapeutic interventions that slow the progression of amyotrophic lateral sclerosis (ALS). ALS is a disorder with heterogeneous onset, which then leads to common final pathways involving multiple neuronal compartments that span both the central and peripheral nervous system. It is believed that excitotoxic mechanisms might play an important role in motor neuron death in ALS. However, little is known about the mechanisms by which excitotoxicity might lead to the neuromuscular junction degeneration that characterizes ALS, or about the site at which this excitotoxic cascade is initiated. Using a novel compartmentalised model of site-specific excitotoxin exposure in lower motor neurons *in vitro*, we found that spinal motor neurons are vulnerable to somatodendritic, but not axonal, excitotoxin exposure. Thus, we developed a model of somatodendritic excitotoxicity *in vivo* using osmotic mini pumps in Thy-1-YFP mice. We demonstrated that *in vivo* cell body excitotoxin exposure leads to significant motor neuron death and neuromuscular junction (NMJ) retraction. Using confocal real-time live imaging of the gastrocnemius muscle, we found that NMJ remodelling preceded excitotoxin-induced NMJ degeneration. These findings suggest that excitotoxicity in the spinal cord of individuals with ALS might result in a die-forward mechanism of motor neuron death from the cell body outward, leading to initial distal plasticity, followed by subsequent pathology and degeneration.

## INTRODUCTION

Amyotrophic lateral sclerosis (ALS) is the predominant form of motor neuron disease (MND) and is one of the most devastating neurodegenerative disorders, with no effective treatment or cure currently available (Talbot, 2014). In ALS, the selective death of upper and lower motor neurons of the cortico-motor-neuronal system results in progressive and terminal atrophy of muscle. As a result, the majority of affected individuals die from respiratory failure within 2–5 years of diagnosis (Rowland and Shneider, 2001). Although 10% of ALS cases have an inherited genetic mutation (Kiernan et al., 2011; Ajroud-Driss and Siddique, 2014), the majority of cases are sporadic. ALS is a multifactorial disease involving RNA dysfunction, protein misfolding and aggregation, ER stress, axonal disruption and excitotoxicity (Gibson and Bromberg, 2012).

Excitotoxicity is caused by over-stimulation of neuronal glutamate receptors, leading to neuronal dysfunction and ultimately to cellular death (Arundine and Tymianski, 2003). Excitotoxicity has been suggested to be a contributing factor in many neurodegenerative disorders (Mehta et al., 2013), and there is accumulating evidence that excitotoxicity plays a central role in ALS (Talbot, 2014; Vucic et al., 2014). Motor neurons are vulnerable to excitotoxic insults (Bar-Peled et al., 1999; Sun et al., 2006; King et al., 2007; Mitra et al., 2013); individuals with ALS have raised levels of glutamate in their cerebrospinal fluid (Rothstein et al., 1990) and display hyperexcitability of motor neurons, which potentially starts before symptom onset (Pieri et al., 2003; Vucic and Kiernan, 2006; van Zundert et al., 2008; Bae et al., 2013). Adding to this, the astrocytes of affected individuals have an impaired glutamate-recycling ability (Rothstein et al., 1996; Sasaki et al., 2000; Banner et al., 2002). Furthermore, the only drug approved for the treatment of ALS, riluzole, acts on the excitotoxic pathway (reviewed in Morren and Galvez-Jimenez, 2012). However, it is not clear why the systemic application of riluzole has limited therapeutic efficiency, increasing the life span of ALS individuals for only a few months.

The cell bodies of lower motor neurons reside within the central nervous system (CNS) in the spinal cord; their distal axons reside within the peripheral nervous system, innervating skeletal muscle at the neuromuscular junction (NMJ). The degeneration of NMJs is considered a hallmark feature of ALS onset and progression (Rocha et al., 2013). Previous studies have highlighted an under-appreciated capacity for NMJ plasticity prior to this NMJ retraction and synaptic loss. Specifically, degeneration-resistant fibre types in the muscles of mouse models of ALS compensate for NMJ degeneration with collateral sprouting and re-innervation (Frey et al., 2000; Schaefer et al., 2005; Pun et al., 2006). Hence, understanding the mechanisms of compensatory plasticity at the NMJ could aid in determining strategies to limit the denervation characteristic of ALS.

Despite the degeneration of these specialised NMJ structures being a key early pathological feature of ALS, it is unknown whether a peripheral excitotoxic insult or a cell body excitotoxic insult results in NMJ retraction. To investigate the specific site of vulnerability of lower motor neurons, we utilised sophisticated novel models of excitotoxin exposure targeted to either the cell body or the NMJ. These studies identified that the cell bodies, but not the peripheral NMJs, of motor neurons are vulnerable to excitoxicity, suggesting that excitoxicity-directed therapeutics could be more effective if targeted to the lower motor neuron soma located in the spinal cord.

**TRANSLATIONAL IMPACT**

**Clinical issue**

Motor neuron disease (MND) is a collective term for a devastating group of disorders that result in muscle paralysis through the degeneration of motor neurons. Amyotrophic lateral sclerosis (ALS) is the most common form of MND, in which affected individuals generally die from respiratory failure within 2–5 years of diagnosis. The degeneration of the neuromuscular junction is considered a hallmark of ALS onset and progression. There is a desperate need to develop therapeutic strategies for this incurable disease. Previous research has provided extensive evidence of a pivotal role for excitotoxicity in ALS pathophysiology. Despite this, little is known about the mechanisms by which excitotoxicity might lead to the neuromuscular degeneration that characterizes ALS. In addition, the site at which this excitotoxic cascade is initiated has not been definitively identified.

**Results**

In this study, the authors established complementary *in vitro* and *in vivo* models to investigate site-specific excitotoxic insult to lower motor neurons in the spinal cord. The results show that lower motor neurons are vulnerable to somatodendritic, but not axonal, excitotoxin exposure *in vitro*. *In vivo*, cell body excitotoxin exposure leads to significant motor neuron death and neuromuscular junction retraction. Live imaging of the neuromuscular junctions demonstrated that, prior to degeneration, neuromuscular junctions have a capacity for compensatory plasticity following chronic excitotoxin exposure.

**Implications and future directions**

This research suggests that excitotoxicity in the spinal cord of individuals with ALS might result in a die-forward mechanism of motor neuron death from the cell body outward, leading to initial distal plasticity that is followed by subsequent pathology and degeneration. Hence, targeted interventions directed specifically to the cell body in the spinal cord might be efficacious in tackling this devastating disease. Additionally, the two models of motor neuron pathology established in the current study could be used to trial a suite of targeted therapeutics for ALS.

## RESULTS

### The exposure of primary spinal motor neurons to kainic acid *in vitro* induces distal axon degeneration

**Fig. 1.**
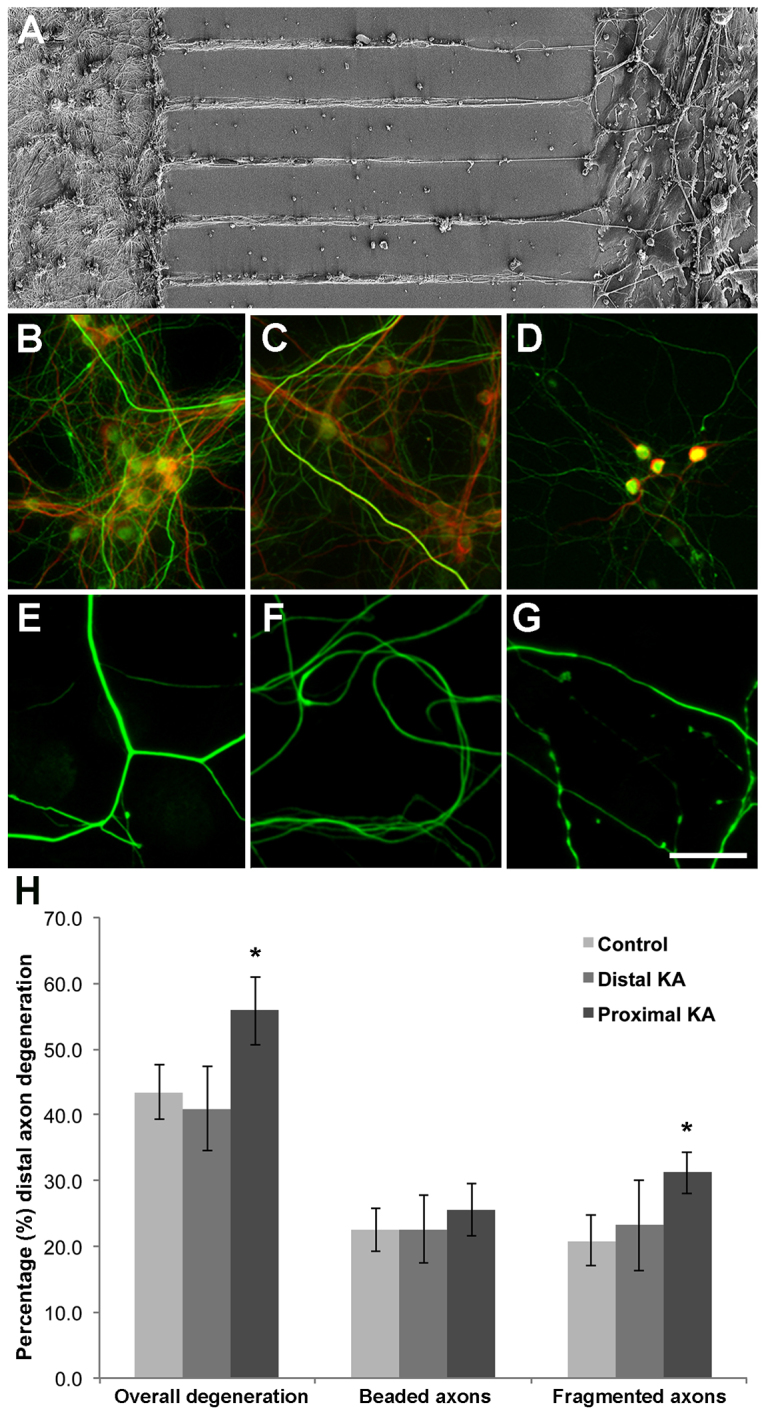
***In vitro* model of site-specific excitotoxicity.** (A) Primary spinal motor neurons were grown in microfluidic chambers. (A) SEM analyses demonstrated primary motor neuron cell bodies (left) spatially separated from C2C12 muscle cells (right) in the distal chamber with interconnecting axons. (B–G) Immunocytochemistry for the cytoskeletal markers that are enriched in dendrites (MAP2, red) and in axons (SMI32, green). (B) Proximal chamber with vehicle control has neurons and continuous neurites immune-positive for both MAP2 (red) and SMI32 (green). (C) The proximal compartment, following treatment of the distal compartment with kainic acid, has neurons and continuous neurites positive for both MAP2 (red) and SMI-32 (green). (D) The proximal chamber, following treatment with kanic acid at the proximal end, demonstrated a loss of immunoreactivity for both MAP2 (red) and SMI32 (green). (E) The distal compartment following treatment with the vehicle control has continuous neurites that are immuno-positive for SMI32 (green). (F) The distal compartment following treatment with kainic acid at the distal end has continuous neurites that are immuno-positive for SMI32 (green). (G) The distal compartment, following treatment with kainic acid at the proximal end, demonstrated axonal degeneration (green). (H) Quantitation of axon degeneration in the distal chamber after treatment with vehicle control, or application of kainic acid (KA) to the distal or proximal compartments. A significant increase in total distal axon degeneration following treatment with kainic acid at the proximal end was seen. Furthermore, there was a significant (*P<0.05) increase in axon fragmentation in the distal compartment following exposure of the proximal compartment to kainic acid. Error bars represent s.e.m. Scale bars 100 μm (A); 50 μm (B–G).

To mimic the cellular interactions that occur *in vivo*, we used microfluidic chambers to construct a compartmentalised co-culture model (Southam et al., 2013). The proximal compartment contained spinal glial cells and the cell bodies of primary spinal cord lower motor neurons from embryonic mice; these motor neuron axons extended through axon channels to the distal compartment that contained skeletal muscle cells (Southam et al., 2013). Scanning electron microscopy (SEM) analyses showed that the cell bodies of the spinal motor neurons were confined to the proximal chamber and that their axons extended from this compartment through interconnecting channels to the distal compartment, which contained C2C12 muscle cells that were confined to this chamber (Fig. 1A). We confirmed the compartmentalisation with immunohistochemistry: spinal motor neurons that were immunoreactive for SMI32 (the neurofilament protein enriched in motor neurons) and MAP2 were present in the proximal compartment of the microfluidic chamber (Fig. 1B); SMI32-positive axons were present in the distal chamber (Fig. 1D).

To investigate the response of spinal motor neurons to site-specific excitotoxicity, 100 μm kainic acid was added to either the distal or proximal compartment of the microfluidic chambers for 24 hours, followed by fixation and analysis. Kainic acid is routinely used as an experimental CNS excitotoxin (Kwak and Nakamura, 1995; Nottingham and Springer, 2003; Mazzone et al., 2010; Mitra et al., 2013) and, unlike glutamate, is not taken up by astrocytes, ensuring a constant level of exposure of the motor neurons to the kainic acid excitotoxin. Application of kainic acid to the distal compartment did not result in changes to either MAP2 or SMI32 labelling in the proximal compartment (Fig. 1C), or SMI32 labelling in the distal compartment (Fig. 1F), relative to the vehicle control treated cultures. However, axons located in the distal compartment demonstrated non-continuous immunolabelling of SMI32 following proximal excitotoxin exposure (Fig. 1G). Quantitation of distal axon degeneration revealed a significant increase in the percentage of degenerating axons (displaying either a beaded or fragmented morphology) following proximal kainic acid exposure, in comparison with vehicle controls (degenerating axons – proximal kainic acid 55.80±2.58% versus vehicle control 43.40% ± 2.08%, *P*=0.0074) (Fig. 1H). Kainic acid application to the distal compartment resulted in no significant distal axon degeneration (41.00±3.22% degenerating axons; *P*>0.05 versus vehicle control treatment) (Fig. 1H). Investigation into the type of axonal damage (fragmentation of the axon versus axonal beading, Hosie et al., 2012) indicated that proximal kainic acid excitotoxicity initiated a significant increase in axonal fragmentation (vehicle control treatment 20.9±1.90%; distal kainic acid 23.30±2.58%; proximal kainic acid 31.20±1.60% fragmented axons, *P*=0.0375 versus vehicle control); however, there was no significant change in axonal beading with either proximal or distal excitotoxin exposure (vehicle control treatment 22.50±1.65%; distal kainic acid 22.60%±2.58; proximal kainic acid 25.60% ± 2.00% beaded axons; *P*>0.05).

### The chronic exposure of lower motor neurons to a proximal excitotoxin triggers dose- and time-dependent motor neuron degeneration

**Fig. 2.**
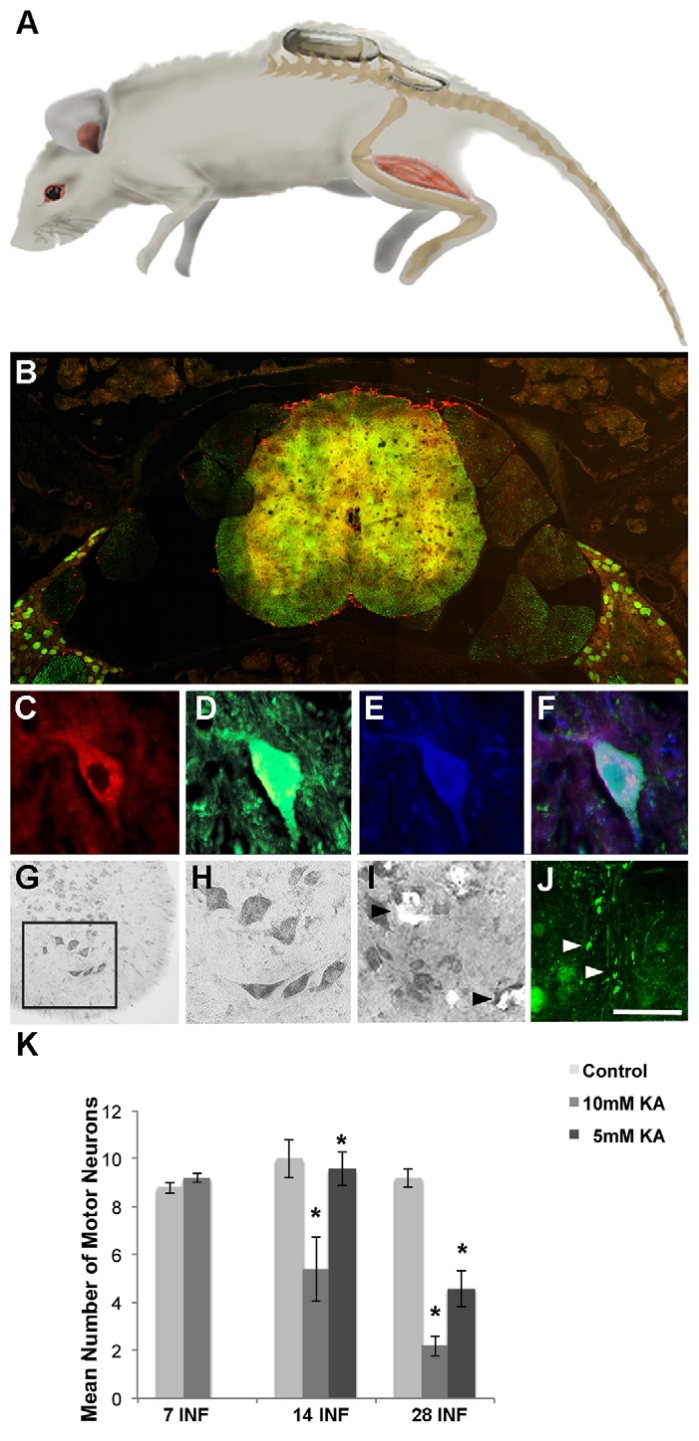
***In vivo* chronic exposure of the spinal cord to kainic acid – proximal effects.** (A) Osmotic mini pumps were placed subcutaneously with the catheter inserted into the subarachnoid space at L5 of YFP-expressing mice. (B) Fluoro-Ruby (red) labelling was present at L4 of the spinal cord of YFP-expressing transgenic mice (green) after 7 days of infusion. (C–F) Fluoro-Ruby (C, red), YFP (D, green) with immunohistochemistry for SMI32 (E, blue) and merged (F), in the anterior ventral horn of the L4 region, demonstrated that kainic acid was being taken up by motor neurons of the spinal cord. (G,H) Toluidine Blue staining following treatment with the vehicle in the anterior ventral horn (H, inset of G) of the spinal cord at L4 on day 28 of the infusion time course. (I) Toluidine Blue staining in the ventral horn of mice treated with an infusion of 10 mM KA for 28 days (arrowheads) demonstrated cell loss. (J) Confocal microscopy demonstrated that there was axonal fragmentation (arrowheads) in the dorsal corticospinal tract after 28 days of infusion. (K) Quantitation of Toluidine-Blue-stained motor neurons in the anterior ventral horn of the spinal cord in the L3 to L4 region after 7, 14 and 28 days of infusion (INF) demonstrated that there were no changes between animals that had been treated with vehicle control and 10 mM kainic acid (KA) for 7 days. There was a significant (*P<0.05) reduction in the mean number of motor neurons at days 14 and 28 in the spinal cords that had been treated with 10 mM kainic acid in comparison with those treated with vehicle control. There was also a significant difference between animals treated with 5 mM and 10 mM kainic acid after 14 and 28 days of infusion. Error bars represent s.e.m. Scale bars: 120 μm (B); 40 μm (C–F); 100 μm (G); 50 μm (H,I); 100 μm (J).

To investigate the effect of chronic excitotoxin exposure on lower motor neuron function, we utilised osmotic mini pumps to provide a continuous infusion of excitotoxin to the lumbar spinal cord. To achieve targeted excitotoxin exposure to the yellow fluorescent protein (YFP)-expressing lower motor neurons innervating the gastrocnemius muscle (Feng et al., 2000), we targeted the L4 region of the spinal cord (Mohan et al., 2014); the mini pump catheter was inserted caudal to the L4 region of the spinal cord (Fig. 2A). To investigate the effect of excitotoxin exposure directly at the somata of lower motor neurons, 5 mM or 10 mM kainic acid was infused into the L3 to L4 region of the spinal cord over a 28-day infusion time course. Fluoro-Ruby tracer was also included in all mini-pump-delivered infusions to ensure correct placement of the catheter in all mice. By 7 days of infusion, the subarachnoid space of the L3 to L4 region had been clearly infused with Fluoro-Ruby (Fig. 2B), demonstrating a constant delivery from the mini pumps to this region. Immunohistochemical labelling of spinal cord sections for the dephosphorylated neurofilament protein SMI32 showed that YFP-expressing SMI32-positive cells in the ventral horn were also positive for Fluoro-Ruby (Fig. 2C–F), indicating that motor neurons had been successfully exposed to the infusions.

After 7 days of infusion, there was no significant difference in the number of motor neurons in the ventral horn of the L3 to L4 spinal cord region between animals that had received the vehicle control (8.80±0.23 motor neurons) and those that had received the 10 mM kainic acid infusion (9.20±0.18 motor neurons, *P*>0.05 versus vehicle control) (Fig. 2K). However, after 14 days of infusion, there was a significant decrease in the number of motor neurons in animals that had received the 10 mM kainic acid infusion (5.40±1.35 motor neurons) in comparison to that of vehicle-treated control animals (10.00±0.78 motor neurons, *P*<0.0001 versus kainic acid infusion) (Fig. 2J). Interestingly, mice that had been treated with a 5 mM kainic acid infusion for 14 days showed no significant difference in the number of motor neurons in comparison with that of vehicle control mice (5 mM kainic acid 9.58±0.70 motor neurons, *P*=0.7085 versus vehicle control), and they retained significantly more motor neurons in the L3 to L4 region at 14 days of infusion than those mice treated with 10 mM kainic acid (*P*<0.0001).

After 28 days of infusion, mice receiving either 5 mM or 10 mM kainic acid infusion showed significantly reduced numbers of motor neuron cell bodies in the L3 to L4 region in comparison with vehicle controls (motor neurons – vehicle control 9.20±0.38, 5 mM kainic acid 4.58±0.76, *P*=0.0003 versus vehicle control; 10 mM kainic acid 2.2±0.42, *P*<0.0001 versus vehicle control) (Fig. 2K). Additionally, animals that had been infused with 5 mM or 10 mM kainic acid for 28 days showed reduced numbers of motor neurons compared with those that had received 5 mM or 10 mM kainic acid for 14 days (*P*<0.0015 and *P*<0.0001, respectively). At 28 days of infusion, there was also a significant difference between animals that had received 5 mM and 10 mM kainic acid (*P*=0.0342). Comparison of the effects of 5 mM and 10 mM kainic acid after 14 and 28 days of infusion suggests a dose-dependent and time-dependent loss of motor neurons in response to proximal excitotoxin exposure. High-power confocal microscopy analyses demonstrated that axonal degeneration was also present in the corticospinal tract at 28 days of infusion (Fig. 2J). No pathology was present at 28 days of infusion in the cortex of treated mice (data not shown).

### The chronic exposure of lower motor neurons to a proximal excitotoxin triggers distal remodeling

**Fig. 3.**
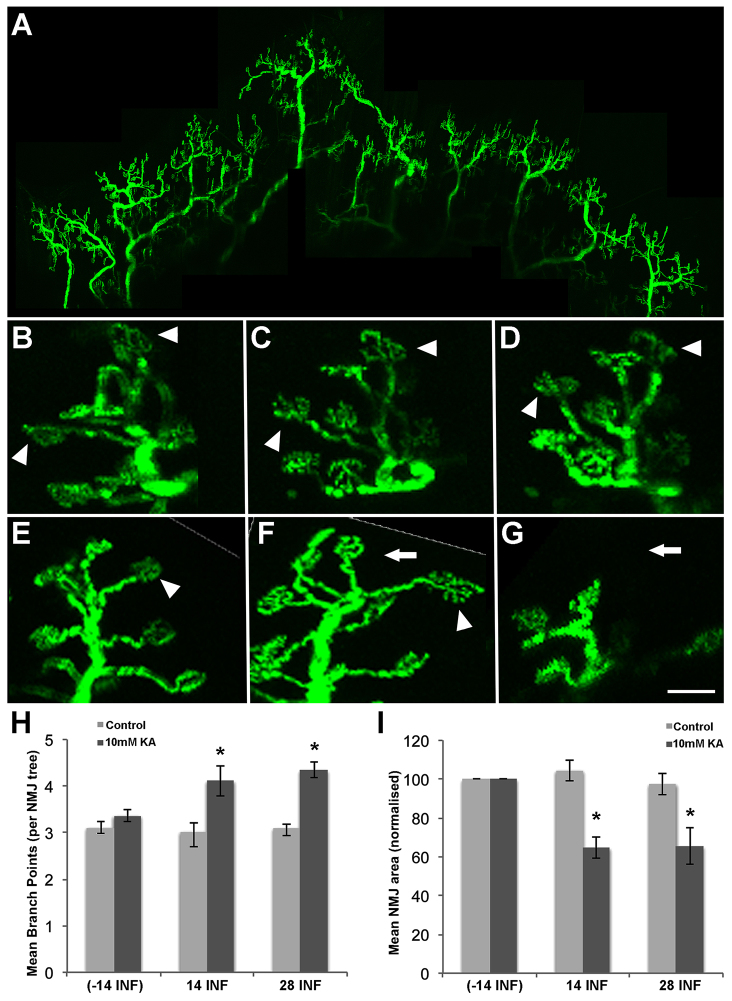
***In vivo* chronic exposure of the spinal cord to kainic acid – distal effects over a live-imaging time course.** (A) Confocal microscopy images of YFP-labelled NMJs in the gastrocnemius muscle. (B–D) NMJ trees of vehicle-control mice were relatively stable (arrowheads) over the imaging time course at days −14 (B), 14 (C) and 28 (D). (E–G) An NMJ tree of mice treated with 10 mM kainic acid over the imaging time course at days −14 (E), 14 (F) and 28 (G) underwent morphological alterations (F; arrowheads) and NMJ loss (G; arrows). (H) There was a significant increase in the number of NMJ branch points at days 14 and 28 for the kainic acid (KA)-treated mice. (I) Quantitation of the mean NMJ area demonstrated a significant (*P<0.05) reduction in the mean NMJ area at both day 14 and day 28 for the KA-treated mice compared with vehicle control. There was no significant difference between the results at days 14 and 28. Error bars represent s.e.m. INF, days of infusion. Scale bars: 500 μm (A); 50 μm (B–G).

To investigate the response of the NMJs in the gastrocnemius muscle to chronic, proximally delivered kainic acid exposure, the gastrocnemius muscle NMJs in mice that had been implanted with kainic-acid-containing osmotic mini pumps (as described above) were repeatedly imaged over an 8-week time course. Imaging was performed before pump insertion (at day −14), then after 14 and 28 days of infusion (Fig. 3). In the animals that had been treated with the vehicle control, the NMJs remained relatively stable (Fig. 3B–D, arrowheads indicate stable and persistent junctions). In the animals that received spinal cord infusion of 10 mM kainic acid, NMJs demonstrated alterations in the morphology of the NMJ trees and in the total junction area (Fig. 3F,G, arrowheads), and NMJs were also lost (Fig. 3F,G, arrow). The number of branch points in the NMJ tree structures was quantified. There were no significant differences in the mean number of branch points between day −14 (3.11±0.12), day 14 (3.02±0.18) and day 28 (3.10±0.08) for vehicle-treated mice. In the mice treated with 10 mM kainic acid, the mean branch point area was significantly increased at day 14 (4.11±0.33) and at day 28 (4.34±0.08, *P*=0.0014) in comparison with that at day −14 (3.36±0.12) (Fig. 3H). In mice that had been treated with the vehicle control, there was no significant (*P*>0.05) difference in the mean NMJ area (normalised) after 14 (104.48±0.18) or 28 days of infusion (97.54±0.079). In mice treated with 10 mM kainic acid, the mean NMJ area was significantly decreased at day 14 (64.60±0.33, *P*=0.073) and at day 28 (65.39±0.16, *P*=0.019) in comparison with the starting NMJ area at day −14. There was no significant difference in the mean NMJ area at day 14 and day 28 in mice receiving 10 mM kainic acid (Fig. 3I).

### The chronic exposure of lower motor neurons to a proximal excitotoxin triggers distal pathology and motor deficits

**Fig. 4.**
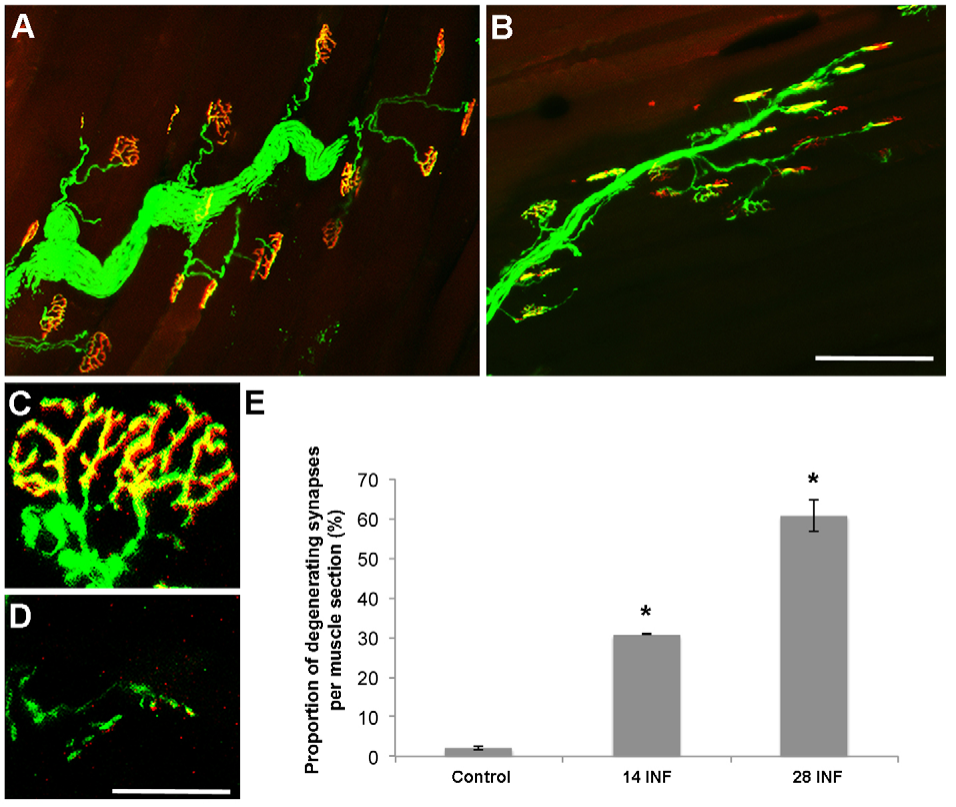
***In vivo* chronic exposure of the spinal cord to kainic acid – distal effects.** (A) After 28 days of infusion (INF), NMJs treated with vehicle control and labelled for YFP (green) and α-bungarotoxin (red) were intact throughout the gastrocnemius muscle. (B) At 28 days of infusion with 10 mM kainic acid, NMJs labelled for YFP (green) and α-bungarotoxin (red) were shrunken. (C,D) NMJs were scored as either intact (C) or degenerating (D). (E) Quantitation of the percentage of degenerating synapses demonstrated a significant (*P<0.05) increase in the percentage of degenerating synapses in the mice that had been infused with 10 mM kainic acid for 14 and 28 days, in comparison with vehicle controls. Error bars represent s.e.m. Scale bars: 100 μm (A,B); 30 μm (C,D).

To investigate the pathological changes occurring in distal motor neuron axons resulting from chronic excitotoxicity in the spinal cord, the NMJs in the gastrocnemius muscle were examined. Gastrocnemius muscle sections with YFP-labelled presynaptic terminals (Fig. 4A,B, green) were counterstained with the postsynaptic stain α-bungarotoxin (Fig. 4A,B, red) so the complete NMJs could be investigated. The NMJs in vehicle-control animals were intact and continuous throughout the endplate zone of the gastrocnemius muscle (Fig. 4A). By day 28, NMJs were frequently shrunken in the mice that had been treated with 10 mM kainic acid (Fig. 4B). To quantify the degree of degenerative pathology occurring over the 28-day time course, the numbers of intact (Fig. 4C) and degenerating (Fig. 4D) synapses were determined. There was a significant increase in the proportion of degenerating synapses relative to intact synapses at day 14 (30.85±0.07%, *P*=0.0161) and at day 28 (60.92±4.01%, *P*=0.0023) in kainic-acid-treated mice in comparison with the vehicle-treated control mice after 28 days of infusion (2.20±0.59%) (Fig. 4E).

**Fig. 5.**
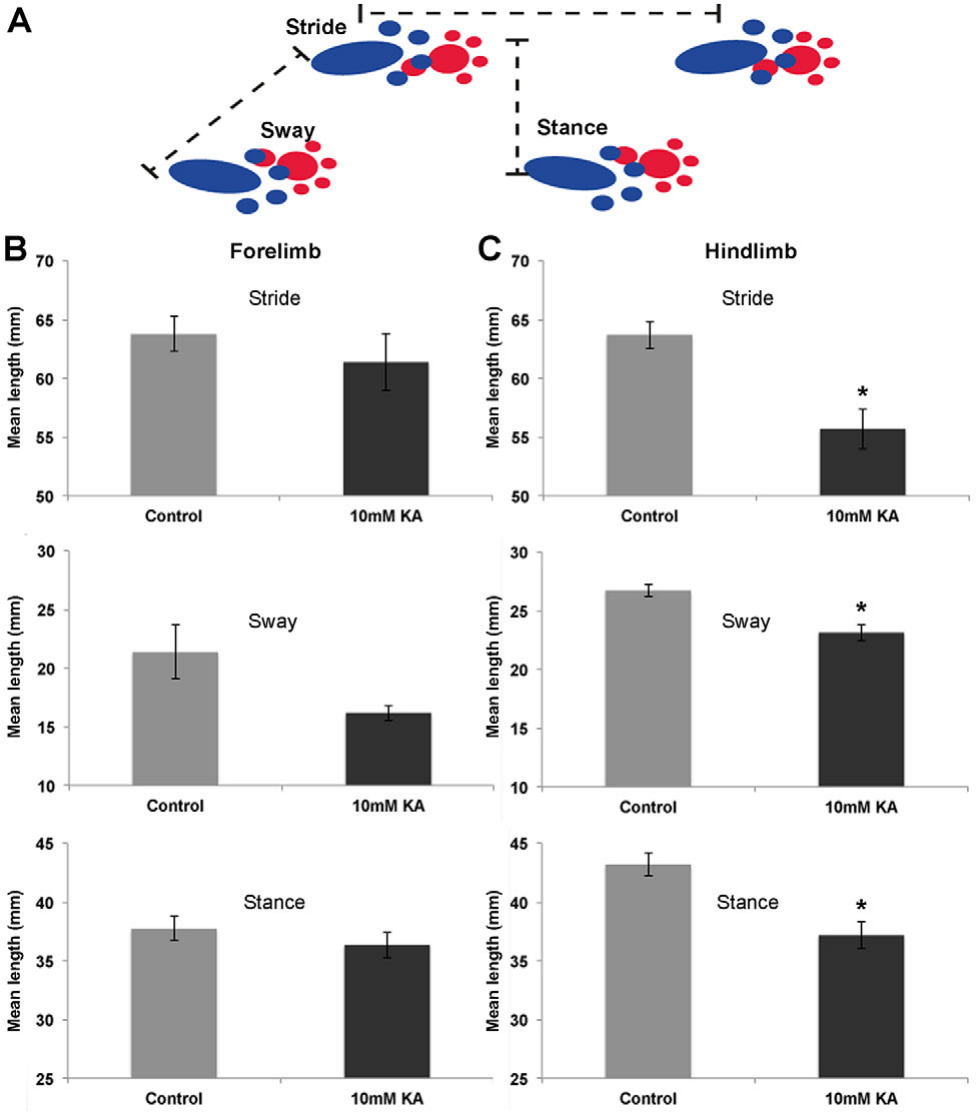
**Forelimb and hindlimb motor function following 28 days infusion.** (A) Motor performance was investigated using analysis of stride, sway and stance lengths for mice that had received 28 days of infusion of kainic acid or vehicle control into the spinal cord. (B) There was no significant difference between the stride, stance and sway lengths of the forelimb stride stance and sway lengths in the mice that had received kainic acid (KA) compared with those of animals receiving the vehicle control. (C) There was significantly (*P<0.05) reduced hindlimb stride, sway and stance length in kainic-acid-treated mice compared with those of animals treated with vehicle control. Error bars represent s.e.m.

The motor function of vehicle-control mice and of mice treated with 10 mM kainic acid was investigated using the footprint test, measuring the ‘stride’, ‘sway’ and ‘stance’ length of both the mouse forelimbs and hindlimbs (Fig. 5A), as described previously (Girirajan et al., 2008). There was no significant (*P*>0.05) difference between the length of the vehicle-control forelimb stride (63.75±1.37 mm), stance (21.36±2.29 mm) or sway (38.48±1.02) and the length of the stride (61.38±2.17), stance (21.36±2.29) or sway (35.55±1.06) of animals that had been treated with kainic acid (Fig. 5B). In the hindlimbs, there was a significant difference in stride length between the animals that had been treated with vehicle control (63.72±1.17 mm) and animals that had received kainic acid (58.39±2.3 mm) (*P*=0.0038) (Fig. 5C). There was also a significant reduction in both sway length (37.19±1.19 mm) (*P*=0.0038) and stance (23.12±0.65 mm) (*P*=0.0004) in those animals that had received the 28-day infusion of kainic acid to the spinal cord compared with those of vehicle controls [43.21±1.02 mm (sway); 26.72±0.55 mm (stance)] (Fig. 5C).

## DISCUSSION

ALS is a devastating disease that is characterised by the selective degeneration of the motor system neurons. However, the clinical presentation and progression rate of ALS can vary (Talbot, 2014) – as such, the site of initiation and the direction of disease propagation have not been conclusively identified. Hyperexcitability of the motor system is a ubiquitous clinical finding in ALS (Kiernan, 2009), with affected individuals typically presenting with muscle fasciculations and cramps (Kleine et al., 2008); this hyperexcitability suggests an excitotoxic mechanism of motor neuron degeneration. Excitotoxicity is a well-recognised potential pathogenic mechanism present in ALS (Talbot, 2014; Vucic et al., 2014), yet it is unknown whether the primary site of excitotoxic damage in ALS is at the cell body or at the distal axon of upper or lower motor neurons. The literature on ALS describes two potential phenomena of motor neuron degeneration: a dying-forward mechanism, where insult to the motor neuron cell body causes cell dysfunction and leads to neuromuscular junction retraction (Vucic and Kiernan, 2006; Kiernan and Petri, 2012); and a dying-back mechanism, where insult to the distal motor neuron axon causes axonal dysfunction and NMJ retraction, potentially prior to cell loss (Fischer et al., 2004; reviewed in Moloney et al., 2014). Our present study is the first, to date, to investigate the effects of either proximal or distal excitotoxin exposure on spinal motor neuron health. In the present study, we used both *in vitro* microfluidic techniques with primary motor neurons and an *in vivo* model of chronic kainic acid exposure in order to demonstrate that exposure of the cell body alone to an excitotoxin (kainic acid) is sufficient to lead to substantial motor neuron death and NMJ retraction. Additionally, we showed that neuromuscular junction plasticity and remodelling preceded this excitotoxin-induced degeneration. Our studies indicate that excitotoxin-exposed lower motor neurons exhibit propagation of dysfunction in a dying-forward mechanism, with exposure of the cell body to an excitotoxin causing NMJ retraction and degeneration, but distal excitotoxin exposure having little effect on the cell body.

### Excitotoxin exposure at the cell body of lower motor neurons results in a dying-forward pathology with ALS-like features

In the current study, the *in vitro* localised application of kainic acid to either the distal axons of motor neurons or to the motor neuron soma was achieved through culture in fluidically isolated chambers. Previous research has identified that cultured motor neurons are vulnerable to extrinsic excitotoxin exposure, resulting in axonal dysfunction and apoptotic neuronal death (Bar-Peled et al., 1999; King et al., 2007). In the present study, we expand on these findings and show that *in vitro* exposure of lower motor neuron cell bodies, but not distal axons, to excitotoxic kainic acid results in substantial fragmentation and degeneration of the distal axons. Our results suggest that excitotoxicity triggers a dying-forward mechanism, where the insult to the lower motor neuron cell body results in distal degeneration.

We have additionally shown that specific application of kainic acid to the spinal cord *in vivo* causes not only the loss of motor neuron cell bodies but also neuromuscular junction remodelling and degeneration. These experiments indicate that the exposure of lower motor neuron cell bodies to an excitotoxic stimulus results in a dying-forward mechanism of excitotoxic damage, characterised by the development of ALS-like features: selective motor neuron loss, neuromuscular junction remodelling, and distal axon damage and neuromuscular junction retraction. Our results concur with those of Sun and colleagues (Sun at al., 2006) and of Mitra and colleagues (Mitra et al., 2013), who showed that *in vivo* chronic excitotoxin exposure can lead to neuronal cell loss in the rat. Furthermore, our present study has characterised the distal NMJ degeneration arising from excitotoxic signals at the proximal soma. Our data are in agreement with those from a previous study in mice, which demonstrate that exposure of retinal ganglion cell somas to glutamate results in a degeneration of the distal axon (Saggu et al., 2008). Thus, the dying-forward propagation of excitotoxin-induced motor neuron damage appears to be consistent between mice and rats.

Interestingly, the chronic *in vivo* application of kainic acid over a 28-day time course did not result in complete motor neuron death and functional loss. This is indicative of the presence of a potentially excitotoxin-resistant population of motor neurons. Previous studies have identified that the motor neurons that are resistant to ALS pathology are also resistant to excitotoxicity (Brockington et al., 2013; Saxena et al., 2013). Of note in the current investigation, the proportion of motor neuron cell bodies lost after 14 days of exposure to excitotoxin was greater than the proportion of neuromuscular junctions lost at the same time point, compared to their respective baselines. Additionally, our live-imaging studies demonstrated remodelling of NMJ synaptic trees and an increase in synaptic branch points over the 28-day kainic acid infusion time course. Taken together, these data indicate that compensatory plasticity might occur in response to excitotoxic damage at the cell body. It is well documented that distal motor neuron segments have the ability to remodel and make new junctions through collateral branching, and previous studies in mouse models of familial ALS have demonstrated that subtypes of motor neurons demonstrate compensatory plasticity (Frey et al., 2000; Schaefer et al., 2005; Pun et al., 2006). Our investigations suggest that this compensatory plasticity might be occurring in the neurons that are potentially resistant to excitotoxicity. Understanding the mechanisms that enable any excitotoxin-resistant motor neurons to resist apoptosis and undergo compensatory plasticity is an intriguing avenue for future investigations; confirming and harnessing these potential mechanisms could present novel therapeutic targets for the treatment of ALS.

It has been hypothesised that excitoxicity in ALS arises from two potential mechanisms: (1) raised extracellular glutamate or (2) indirect excitotoxicity, occurring when extracellular levels of glutamate are normal but neurons become more sensitised to normal glutamate levels. Indeed, motor neurons show an inherent susceptibility to excitotoxicity due to their unique intrinsic handling of Ca^2+^, which is reliant heavily upon mitochondria (Ince et al., 1993; Parone et al., 2013), and to their decreased expression of the Ca^2+^-impermeable GluR2 glutamate receptor subunit (Van Damme et al., 2002), allowing Ca^2+^ influx into motor neurons. A recent study in the SOD1 mouse model of ALS demonstrated that motor neurons are not intrinsically excitable prior to dysfunction, further implying that the excitotoxicity-causing events must be extrinsic to the motor neuron (Delestrée et al., 2014). Furthermore, levels of the main inhibitory neurotransmitter γ-aminobutyric acid (GABA) are reduced in individuals with ALS, indicating reduced interneuron inhibition at the cell body (Turner and Kiernan, 2012). Thus, it is clear that many possible sources of cell body excitotoxicity could contribute to ALS; our data in the present study certainly indicate that excitotoxic stimulation in the murine spinal cord leads to ALS-like cell loss, NMJ remodelling, NMJ retraction and functional deficits.

### Possible differences between upper and lower motor neuron axons in the response to distal axon excitotoxicity

In the present study, no distal degeneration was observed upon direct exposure of lower motor neuron distal axons to the excitotoxic stimulus *in vitro*, indicating that distal axons from lower motor neurons are not susceptible to direct excitotoxicity. However, it is interesting to note that we observed some corticospinal tract pathology after 28 days of infusion of kainic acid into the spinal cord. It is not clear whether this corticospinal tract pathology was due to a direct effect of kainic acid excitotoxin exposure on the distal axons of upper motor neurons, or due to retraction of the upper motor neuron distal axons upon the loss of their targets. If the latter were true, the corticospinal tract pathology could indicate that upper motor neurons are affected by dying-back mechanisms of degeneration triggered by death of the lower motor neurons. However, if the former were true, this would indicate that the distal axons of upper motor neurons, but not of lower motor neurons, are susceptible to direct excitotoxicity. This remains an intriguing research question for further investigation, potentially by using live imaging; however, limited access to the spinal cord due to the placement of the osmotic pump prohibits this approach in the current model.

The possibility of differential susceptibility to excitotoxicity between upper and lower motor neuron distal axons is intriguing, and might be related to the distribution of glutamate receptors on the upper and lower distal axons and their respective synapses. The extra-synaptic expression of glutamate receptor subtypes, specifically along myelinated axons (Passafaro et al., 2001; Tovar and Westbrook, 2002; Kane-Jackson and Smith, 2003; van Zundert et al., 2004), suggests a wider role for glutamate than solely as an inter-neuronal excitatory transmitter (Araque and Perea, 2004). Excitotoxicity within the white matter occurs in a number of degenerative conditions – including glaucoma (Saggu et al., 2008) and multiple sclerosis (Pitt et al., 2003) – and it is also common following injury (reviewed in Lau and Tymianski, 2010). The white matter tracts are distinctly vulnerable to degeneration in ALS, suggesting that myelinated axons in the white matter tracts are vulnerable to excitotoxicity owing to their expression of extra-synaptic glutamate receptor subtypes (Ozdinler et al., 2011; King et al., 2012). However, the majority of this regional vulnerability research has focused within the white matter of the CNS. Lower motor neurons differ from upper motor neurons in both their spatial location, with their proximal cell bodies residing in the CNS (spinal cord) but their distal NMJs residing within the peripheral system (on skeletal muscle), and potentially the distribution of their extra-synaptic glutamate receptors.

In lower motor neurons, previous studies have identified postsynaptic ionotrophic glutamate receptors at the mouse NMJ (Mays et al., 2009) and at the pre-synapse in the lizard NMJ (Walder et al., 2013), and pre-synaptic ionotrophic glutamate receptor organisation occurs at the rat NMJ during re-innervation by glutamatergic neurons following transection injury (Brunelli et al., 2005), suggesting a potential role for glutamate at the NMJ. However, lower motor neurons have no known functional extra-synaptic glutamate receptor expression. This could explain the lack of direct excitotoxin-mediated damage at the lower motor neuron distal axons in our *in vitro* cell culture model in the current study, compared with the observation of corticospinal tract pathology in upper motor neurons after distal axon exposure to infusions of kainic acid into the spinal cord. Understanding any differential mechanisms of degeneration between upper and lower motor neurons might help to elucidate the key pathogenic mechanisms at work in ALS.

### Conclusions

In summary, these investigations indicate that raised central levels of excitotoxic stimuli could be a primary mechanism involved in the degeneration of motor neurons, and therefore indicate that excitotoxicity could account for the selective motor neuron death observed in ALS. Understanding excitotoxic mechanisms of neuronal cell loss and NMJ degeneration is a vital step in the pathway to developing potential new therapeutic interventions for ALS. The data presented in this current study suggest that targeted interventions, directed specifically at the cell body, might be more efficacious in tackling this devastating disease.

## MATERIALS AND METHODS

### *In vitro* model of site-specific excitotoxin exposure

Microfluidic chambers (Xona Microfluidics) were prepared for co-cultures as previously described (Southam et al., 2013). Each chamber (microfluidic device with coverslip) was filled with 2.5 ng/ml of laminin in 0.001% poly-L-lysine on one side (proximal compartment) and 2.5 ng/ml laminin in 0.01% collagen in the other side (distal compartment). Chambers were incubated overnight at room temperature, substrates were then removed and the chambers were filled with neuron initial medium (Neurobasal supplemented with 10% heat-inactivated fetal calf serum, 2% B27 neuronal supplement, 1% antibiotic-antimycotic, 0.5 μM L-glutamic acid and 2.5 nM L-glutamine) for 2 hours before the addition of motor neurons.

Primary *Mus musculus* (mouse) motor neuron cultures were prepared from the spinal cords of embryonic day 13.5 (E13.5) C57Bl/6 embryos, purified on an Optiprep™ gradient (1.06 g/l, Sigma) and plated at 8.5×10^4^ cells per prepared microfluidic chamber (Southam et al., 2013). Motor neurons were maintained in neuron initial medium until 7 days *in vitro* (DIV), and neuron subsequent medium (Neurobasal supplemented with 2% B27, 1% antibiotic-antimycotic, and 2.5 nM L-glutamine) thereafter. C2C12 myoblast cells (American Type Culture Collection) were maintained in Dulbecco’s modified Eagle’s medium supplemented with 10% non-heat inactivated fetal calf serum and 1% antibiotic-antimycotic) at less than 70% confluence. Cells for co-culture were maintained at passage 12 or below. C2C12 myoblasts were added to the distal compartments of microfluidic chambers when motor neurons were 4-days old and maintained in neuron subsequent medium supplemented with 10% horse serum for 2 days, followed by neuron subsequent medium supplemented with 7% non-heat inactivated fetal calf serum for 3 days to promote differentiation. Distal compartments were maintained in serum-free neuron subsequent medium thereafter.

After motor neurons had been cultured for 9 DIV, co-cultures were exposed to 100 μM kainic acid in either the proximal or distal compartment for 24 hours. Fluidic isolation of the treated compartment was achieved by maintaining the fluid volume of the treated compartment at a level lower than that of the untreated compartment (Hosie et al., 2012). For immunocytochemistry analyses, primary antibodies (SMI32, 1:2000, Sternberger Monoclonals; MAP2, 1:1000, Millipore) were incubated in 0.3% Triton X-100 (Sigma) in 0.1 M PBS overnight. Cells were thoroughly washed in 0.1 M PBS and incubated with species-specific AlexaFluor secondary antibodies (1:1000, Molecular Probes^®^) for 2 hours. Axonal pathology images of the distal compartment of each coverslip were captured using a Leica (DM LB2) fluorescence microscope fitted with a cooled CCD camera. Axons were assessed within a 50-μm^2^ grid superimposed on each image (ImageJ) and counted as either whole, beaded (distinguishable swellings connected by sections of axon) or fragmented (disconnected swellings). Overall degeneration was calculated as the percentage of axons that were beaded or fragmented.

### Scanning electron microscopy

Cultures for SEM analysis were fixed in 4% paraformaldehyde with 2.5% glutaraldehyde. Microfluidic chambers were removed, and the cultures were dehydrated in an ethanol gradient and stored in acetone. Coverslips were critical-point dried, sputter coated with platinum and examined using a Hitachi SU-70 field emission scanning electron microscope.

### *In vivo* model of site-specific excitotoxin exposure

All experimental procedures involving animals were approved by the Animal Ethics Committee of the University of Tasmania and are in accordance with the NHMRC Australian Code of Practice for the Care and Use of Animals for Scientific Purposes, 2013. *Mus musculus* (mice) were housed in standard conditions (20°C, 12–12 hours light-dark cycle) with access to food and water *ad libitum*. Animals were monitored daily for signs of illness and stress. For targeting the spinal cord, osmotic mini pumps (Azlet^®^ model 1004; infusion rate of 0.11 μl per hour for 28 days) fitted with polyethylene catheter tubing were filled with 5 and 10 mM kainic acid (Sigma) in artificial cerebrospinal fluid (aCSF:125 mM NaCl, 5 mM KCl, 10 mM D-glucose, 10 mM HEPES, 2 mM CaCl_2_, 2 mM MgSO_4_ pH 7.4; all from Sigma). Vehicle control pumps were loaded with aCSF. To ensure appropriate placement of the catheters and focused delivery of all solutions, Fluoro-Ruby (0.01 μm; Fluorochrome, LLC) was added to both vehicle-control and excitotoxin pumps.

Previous investigations in the rat have demonstrated that a single 1 μg injection of kainic acid directly into the ventral horn resulted in excitotoxic cell loss (Mitra et al., 2013). Furthermore, using osmotic mini pumps in the rat, 3.8 μg per 24 hours resulted in loss of hindlimb function and cell loss by 4 weeks (Sun et al., 2006). For proximal excitotoxin exposure in the current study in mice, 10 mM and 5 mM kainic acid was applied to the subarachnoid space of the L3 to L4 region of the spinal cord, which was equivalent to 5 μg and 2.5 μg per 24 hours, respectively, this dosage was chosen based upon the previous studies in rats (Sun et al., 2006). Osmotic pumps were prepared 48 hours before surgery and equilibrated in 9% sterile saline at 37°C, as previously described (Nakamura et al., 1994).

Thy-1-YFP mice [Jackson Laboratory B6;CBA-Tg(Thy1-YFP)GJrs/GfngJ] were used for this study, and adult male mice at ~12 weeks were anaesthetised with a ketamine (120 mg/kg) and xylazine (10 mg/kg) cocktail, and then prepared for surgery. The L5 vertebra was partially laminectomised and the catheter was inserted 3 mm rostral into the spinal cord to terminate at L4. The catheter was superficially fixed in place with Vetbond™ (3M™), the osmotic pump was placed subcutaneously along the spine and the incision site was sutured. For analgesia, mice were given Temgesic (0.01 mg/kg) for 72 hours post-surgery.

### Immunohistochemistry and analysis

Animals were terminally anaesthetised (pentabarbitone sodium, 140 mg/kg), over a time course of infusion up to 28 days, and then perfused with 4% paraformaldehyde. The L3 to L5 region of the spinal column was dissected and placed into a decalcification solution (5% nitric acid, 0.05% urea, both from Sigma, in MilliQ^®^) overnight. The spinal columns were then washed with 0.1 M phosphate buffered saline (PBS) and placed into a series of sucrose solutions (4%, 18% and 30%, Sigma) in 0.1 M PBS. Spinal cord sections were serially cut into 20 μm sections and mounted onto superfrost™ plus slides (Thermo Scientific) for cell body quantitation, and were cut into 60 μm sections for free floating immunohistochemistry. The L5 region was used to check for correct catheter placement. For Toluidine Blue staining, sections were incubated in 0.05% Toluidine Blue in 0.1 M PBS and then thoroughly washed. Gastrocnemius muscles were dissected, placed into a series of sucrose solutions (4%, 18% and 30%, Sigma) in 0.1 M PBS for cryo-protection and sectioned at 80 μm.

For immunohistochemistry, sections were incubated with primary antibodies (SMI32, 1:2000, Sternberger Monoclonals) diluted in 0.3% Triton X-100 (Sigma) in 0.1 M PBS and incubated overnight. Sections were thoroughly washed in 0.1 M PBS and incubated with species-specific AlexaFluor secondary antibodies (1:1000, Molecular Probes^®^) for 2 hours. Muscle sections were stained with α-bungarotoxin (1:100, Molecular Probes^®^) in 0.1 M PBS for synapse quantitation. Images were captured on a Zeiss LSM 510 confocal microscope using Zen software. Z-stacks were flattened in ImageJ and processed in ImageJ and Photoshop (Adobe).

### Repeated live imaging of gastrocnemius muscle

Mice were anaesthetised with the ketamine (120 mg/kg) and xylazine (10 mg/kg) cocktail described above, and imaging of the lateral gastrocnemius muscle was performed essentially as described by Wigston (Wigston, 1990) with modifications. Briefly, mice were placed on a custom-designed heated stage that restrained the hindlimbs to avoid movement artefacts. The lateral surface of the right hind leg was shaved and a small incision was made in the skin overlying the right lateral gastrocnemius muscle, and the muscle was exposed. Images were captured on a Zeiss LSM 510 confocal microscope using a 488-nm confocal laser, 10× 0.3 NA objective with Zen software. Following imaging of NMJs, animals were removed from the microscope, the incisions were sutured and the animals allowed to recover. Fourteen mice (seven receiving vehicle control, seven receiving 10 mM kainic acid) were imaged over a 6-week time course on days −14, 14 and 28, −14 corresponds to 14 days before the pump insertion, day 0 being the day that the pumps were inserted. Z-stacks were analysed by using ImageJ.

### Footprint test

To obtain footprints, the hind- and forefeet of the mice were coated with two different nontoxic paints, respectively. The animals were then allowed to walk along a 50-cm-long, 10-cm-wide runway (with 10-cm-high walls) into an enclosed box (Girirajan et al., 2008). All mice were given two training runs and one test run. After completion, mice were transcardially perfused.

### Statistical analysis

Student’s *t*-tests and one-way and two-way analysis of variance with or without repeated measures (ANOVAs), performed in GraphPad Prism Software, were used where appropriate. Post-hoc comparisons were performed using Bonferroni’s correction for multiple comparisons. *P*<0.05 was considered significant. Data is reported ± standard error of the mean (s.e.m.).
